# MicroRNA‐200a suppresses prostate cancer progression through BRD4/AR signaling pathway

**DOI:** 10.1002/cam4.2029

**Published:** 2019-02-19

**Authors:** Han Guan, Zonghao You, Can Wang, Fang Fang, Rui Peng, Likai Mao, Bin Xu, Ming Chen

**Affiliations:** ^1^ Department of Urology The First Affiliated Hospital of Bengbu Medical College Bengbu China; ^2^ Department of Urology Affliated Zhongda Hospital of Southeast University Nanjing China; ^3^ Department of Immunology Bengbu Medical College Bengbu China; ^4^ Department of Graduate School Bengbu Medical College Bengbu China; ^5^ Department of Urology The Second Affiliated Hospital of Bengbu Medical College Bengbu China

**Keywords:** androgen receptor, BRD4, miRNA, prostate cancer, signaling pathway

## Abstract

Prostate cancer is still considered a significant health care challenge worldwide due in part to the distinct transformation of androgen‐dependent prostate cancer (ADPC) into treatment‐refractory castration‐resistant prostate cancer (CRPC). Consequently, there is an urgent need to explore novel molecular mechanisms underlying treatment resistance in ADPC. Although numerous studies have alluded to the role of miR‐200a in several cancers, the biological significance of miR‐200a in prostate cancer remains unknown. After performing microarray analysis and reanalysis of the publicly available Memorial Sloan Kettering Cancer Center dataset, miR‐200a expression was found higher in ADPC tissues and its expression was positively associated with survival of CRPC patients. In vitro studies showed that miR‐200a overexpression in CRPC cells markedly suppressed cellular proliferation and facilitated apoptosis. In vivo studies indicated that overexpression of miR‐200a inhibited growth and metastasis of prostate cancer. The luciferase reporter assay demonstrated that *BRD4* is a direct target gene of miR‐200a and it could reverse miR‐200a‐mediated biological effects in prostate cancer cells. Most importantly, our findings indicated that miR‐200a suppresses the progression of CRPC by inhibiting the activation of BRD4‐mediated AR signaling. This finding provides the foundation for the development of more personalized therapeutic approaches for CRPC patients.

## 1 INTRODUCTION

Prostate cancer, with its pathological and clinical heterogeneity, has been the second most frequently diagnosed cancer in men and ranked as the fourth most common malignancy in the world.^1^ According to an updated estimate in the US, prostate cancer has the highest incidence amongst male tumors in 2018, accounting for 19% of male malignant tumors, while the mortality rate ranks second.^2^ Prostate cancer is initially androgen dependent and sensitive to androgen deprivation therapy.^3^ However, within 2 years, most prostate cancer (PCa) cases become insensitive to androgen deprivation and are termed castration‐resistant.^4^ Therefore, exploring new molecular mechanisms, as well as novel molecular biomarkers is imperative for the development of personalized therapeutic approaches for the diagnosis, prognosis, and treatment of PCa.

MicroRNAs (miRNAs, or miRs) consist of a series of short (18‐22 nucleotides), noncoding, single‐stranded RNA molecules. miRNAs can selectively bind to the 3'untranslated region (3' UTR) of downstream target mRNAs, promoting their degradation. miRNAs can also impede biosynthesis of specific proteins, and subsequently regulate multiple physiological processes.^5^, ^6^ Also, accumulating evidence shows that dysregulation of miRNAs can have tumor‐suppressive or oncogenic effects in the development and progression of several cancers, including prostate cancer, by regulating tumor growth, metastasis, and epithelial to mesenchymal transition (EMT).^7^, ^8^, ^9^ It has been reported that miR‐133a and miR‐150 inhibit the growth of metastatic prostate cancer by regulating MAP3K12 expression or by activating PI3K/AKT signaling. Oncogenic factors, such as miR‐203, miR‐410, and miR‐34a, have been identified as carcinogenic factors that promote proliferation of PCa cells.^10^, ^11^, ^12^ Notably, miR‐200a, a member of the miR‐200 family, has been shown to participate in several biological processes that regulate the progression of tumors.^13^, ^14^, ^15^, ^16^, ^17^, ^18^ However, the role of miR‐200a in prostate cancer development and progression has not been investigated.

After using miRNA chip expression spectrum detection technology and analyzing the miRNA expression profile of prostate cancer patients in the Memorial Sloan Kettering Cancer Center (MSKCC) database, we found that there were differences in the expression of miR‐200a in androgen‐dependent prostate cancer (ADPC) tissues and castration‐resistant prostate cancer (CRPC) tissues. Quantitative Real‐Time Polymerase Chain Reaction (qRT‐PCR) confirmed that miR‐200a was significantly lower in cancer tissues from CRPC patients. Statistical analysis also revealed that miR‐200a is an independent risk factor for the prognosis of prostate cancer patients.

Therefore, we conducted a series of cellular functional experiments by overexpressing miR‐200a in prostate cancer cells and observing the effect on proliferation, invasion, colony formation, and apoptosis of prostate cancer cells. We also found that bromodomain containing protein 4 (*BRD4*) is a potential downstream target gene of miR‐200a, and the association between miR‐200a and BRD4 was verified by western blot analysis. Therefore, the results of our study suggest that miR‐200a acts as an anti‐oncogenic factor to inhibit the progression of CRPC by suppressing BRD4‐mediated androgen receptor (AR) signaling.

## 2 MATERIALS AND METHODS

### 2.1 Patients and tissue samples

Twenty tissue samples were obtained from PCa patients during surgery at the Affliated Zhongda Hospital of Southeast University (Nanjing, China) including 10 ADPC tissues after early radical prostateectomy and 10 tissues from CRPC patients who suffered from transurethral resection of the prostate (TURP). The age of the patients ranged from 64 to 79 years (average age: 66 years). Among the ADPC patients, eight patients were diagnosed with ADPC stage II and two patients with stage III; seven patients had Gleason score <7; two patients with Gleason score =7, and one patient with Gleason score >7. Castration‐resistant prostate cancer specimens are derived from 10 patients with CRPC, of whom the serum prostate‐specifc antigen (PSA) levels continued to increase maxima during androgen‐deprivation therapy. The clinical stage of patients with CRPC were all in stage IV and all patients with a Gleason score >8. The clinical samples were obtained during TURP because of urinary retention. For each specimen, a portion of tumor tissue was confirmed as staining for PSA and only the samples with >60% tumor involvements were included in the study. The specimens were routinely fixed with polyformaldehyde and embedded with paraffin wax for routine clinical pathological analysis and subsequent experimental research. Informed consent was obtained from the included patients. All procedures performed in this study were approved by the Ethics Committee of the Affliated Zhongda Hospital of Southeast University.

### 2.2 Cell culture

The LNCaP and C4‐2B PCa cell lines were obtained from the Urological Institute of the Affliated Zhongda Hospital of Southeast University. LNCaP cells were maintained in the medium of RPMI‐1640 (Gibco, Thermo Fisher Scientific, Waltham, MA, USA) supplemented with 10% fetal bovine serum and antibiotics; C4‐2B cells were cultured in DMEM/12 medium (Gibco, Thermo Fisher Scientific) supplemented with 10% fetal bovine serum and antibiotics. All cell lines were maintained at 37°C in a humidified chamber supplemented with 5% CO2.

### 2.3 Oligonucleotide and plasmid transfection

Based on miRBase database, miR‐200a mimic, negative control of miRNA (miR‐NC), anti‐miR‐200a oligos (anti‐miR‐200a), and negative control anti‐miRNA (anti‐NC) were obtained from GenePharma (Shanghai, China). Short interfering RNA(siRNA) against BRD4 (si‐BRD4) and negative control siRNA with nonspecific sequences (si‐NC) were all purchased from Santa Cruz Biotechnology (Santa Cruz, CA, USA). For cell transfection, LNCaP and C4‐2B cells were seeded in 6‐well plates and the transfection was performed using Lipofectamine 3000 (Thermo Fisher) following the manufacturer's protocol. At 48 hours after transfection, the cells were used in the MTT (3‐(4,5‐dimethyl‐2‐thiazolyl)‐2,5‐diphenyl‐2‐H‐tetrazolium bromide, Thiazolyl Blue Tetrazolium Bromide), transwell, colony formation, and western blotting assays.

### 2.4 Stable transfection

Reverse complement sequence of miR‐200a was synthesized and inserted into the AgeI/EcoR1 site of GV209 vector (GeneChem, Shanghai, China) to construct a vector expressing miR‐200a named LV‐miR‐200a. The GFP vector was used for control. The viruses were used to infect C4‐2B cells in the presence of polybrene. The effciency of miR‐200a overexpression was determined by fluorescence microscopy.

### 2.5 Colony formation assay

Cell proliferation was evaluated based on colony numbers via the colony formation assay. In brief, LNCaP and C4‐2B cells were seeded in 6‐well plates at a density of 500 cells per well and incubated for 12 days at 37°C in 5% CO2. Next, the cells were washed thrice with phosphate buffer solution(PBS), fxed with methanol for 15 minutes, and stained with 300µL of 0.1% crystal violet for 20 minutes at room temperature. Colonies containing more than 50 cells were counted by the ImageJ 2X software.

### 2.6 Transwell assays

For invasion assays, transfected‐PCa cells were diluted to the final concentration of 5.0 × 10^6^/mL. Postdiluted PCa cells in 100 μl serum‐free medium were seeded in the top chamber of 24‐well transwell units which were precoated with Matrigel (BD Pharmingen, Franklin Lake, NJ, USA) while the bottom chambers were filled with RPMI‐1640 containing 10% FBS. The top chamber was taken out after placing the chamber in the cell incubator for 24 hours at 37°C. Cells in the top chambers were removed and cells that invaded into the bottom chambers were fxed with methanol and stained with 0.2% crystal violet. The cells that migrated to the bottom chamber of the membrane were counted and photographed microscopically.

### 2.7 Cell proliferation assay

For MTT assays, LNCaP and C4‐2B cells were equally plated in 96‐well plates (2000 cells/well) for 24 to 96 hours using Lipofectamine 2000 (Life Technologies, Cambridge, MA, USA) after transfecting miR‐200a or miR‐NC. About 10 μl of MTT (5 mg/ml) was added to the plates at an interval of every 24 hours. After 4 hours of incubation, 200 μL dimethylsulphoxide (DMSO) were added to each well. The optical density was measured at 570 nm with a microplate reader (Bio‐Tek, Winooski, VT, USA).

### 2.8 Apoptosis analysis

Cell apoptosis analysis was performed with Annexin V‐FITC/Propidium Iodide (PI) Apoptosis Detection Kit (Beyotime, China) to detect the cell apoptosis of the LNCaP and C4‐2B cells. Cells in good condition and in logarithmic growth stage were selected first. After transfection for 48 hours, PCa cell lines were stained with 5 µl VFITC and 10 µl PI following the instructions and cultured in the dark at room temperature for 15 minutes. Data analysis was performed using Cell Quest Pro Software (BD Biosciences, CA).

### 2.9 Luciferase reporter assay

After searching the online software programs TargetScan and miRNADA, BRD4 was found to be the potentially downstream target gene among putative genes predicted by the above algorithms. The expression of the target gene of miR‐200a was evaluated in LNCaP cells by a luciferase reporter assay. The fragments of BRD4 3′‐UTR containing either putative miR‐200a seed sequence were synthesized by GeneChem (Shanghai, China). We subcloned wild‐type and mutant BRD4 3′‐UTR into psiCHECK‐2™ vector (Promega, USA) to obtain reporters of psi‐CHECK‐BRD4‐WT and psi‐CHECK‐BRD4‐Mut. LNCAP and C4‐2B cells were seeded in 24‐well plates and cotransfected with either miR‐200a mimics or NC and BRD4‐WT or BRD4‐MUT plasmid using Lipofectamine 2000 reagent (Invitrogen) at 37°C. Cells were collected 48 hours after transfection and analyzed with a Dual‐Luciferase Reporter Assay System (Promega). The luciferase activity was detected by chemiluminescence method. Measurement was conducted thrice to obtain the average value.

### 2.10 RNA extraction and quantitative real‐time PCR (qRT‐PCR)

Total RNA was extracted from PCa cells with TRIzol (Invitrogen™, Thermo Fisher Scientific) following the manufacturer's protocol. cDNA synthesis were performed using total RNA and the PrimeScript RT reagent (Takara, Kusatsu, Japan), and qRT‐PCR was performed on ABI 7300 system (Applied Biosystems, Shanghai, China). miRNA were normalized using GAPDH and the expression of U6 as endogenous controls. PCR results were analyzed using OpticonMonitor3 software (BioRad, Irvine, CA) and relative gene expression was calculated by the 2^−ΔΔCt ^method. The expression of 2^−ΔΔCt^ was the ratio of gene expression between experimental group and control group. Experiments were conducted for three times to obtain the average value.

### 2.11 Western blot analysis

The total proteins were extracted from PCa cells lysed in RIPA buffer according to the manufacturer's instructions and quantified by BCA assay (Beyotime, Shanghai, China). The protein were analyzed by 10% sodium dodecyl sulfate polyacrylamide gels (SDS‐PAGE) and the gels were transferred onto a polyvinylidene fluoride (PVDF) membrane blocked with 5% nonfat dried milk in TBST for 1 hour. Afterward, the PVDF membranes were incubated with specifc primary antibodies overnight at 4°C. After washing three times with TBST buffer, the membranes were then incubated with 1:2000 secondary antibodies for 1 hour and the bands were visualized by an enhanced chemiluminescence (ECL, Millipore, USA). The primary antibodies used in the present study included anti‐BRD4 (1:1000, Abcam, USA), anti‐AR (1:200, Abcam, USA), anti‐AR‐V7 antibody (1:1000, Abcam, USA), and anti‐C‐myc antibody (1:1000, Abcam, USA). GAPDH was used as an internal control.

### 2.12 In vivo tumorigenicity assay

Six‐week old BALB/C nu/nu female mice were purchased from the Shanghai SLAC Laboratory Animals. All animal experiments were approved by the Institutional Animal Care and conducted according to the National Institute of Health Guide for the Care. The availability of laboratory animals was approved by the ethics committee of the Affliated Zhongda Hospital of Southeast University. C4‐2B (4 × 10^6^) cells transfected with LV‐miR‐200a were injected subcutaneously into the oxter flank of nude mice for a 7‐day implantation time during which tumor size was measured at 2‐day interval and tumor volumes were calculated as the follows: V (mm3) = width (mm2) × length (mm)/2. The mice were sacrificed and tumors were dissected and weighed at the end of the experiments.

### 2.13 In vivo metastasis assay and bioluminescence imaging analysis of lung metastasis

C4‐2B (2 × 10^6^) cells transfected with LV‐miR‐200a or LV‐NC for 48 hour were washed and suspended in 0.1 ml PBS and subsequently injected into the lateral tail vein. The injected mice were anesthetized immediately using isoflurance and injected with 150 mg/kg of D‐luciferin (15 mg/ml in PBS) intraperitoneally to demonstrate lung location of PCa cells. After a 7‐week monitoring time, mice were anesthetized and injected with D‐luciferin for 10 minutes as the first time. The nude mice were imaged with a highly sensitive camera in a light‐tight specimen chamber (IVIS200, Xenogen). Living Image software (Xenogen) was conducted to acquire and quantify bioluminescence signals. Bioluminescence imaging was correlated with lung tumor volumes estimated using caliper measurements and a standard formula: 1/6 × width × length × depth.

### 2.14 In situ hybridization (ISH) and immunohistochemical staining (IHC)

MicroRNA in situ hybridization (ISH) Buffer and Controls kit (Exiqon, Vedbaek, Denmark) were used for ISH. The double (5′–3′) digoxigenin (DIG)‐labeled miR‐200a probe and U6 probe were purchased from Boster (Wuhan, China) and ISH was conducted according to the manufacturer's protocol of the microRNA ISH Optimization Kit (Boster, Wuhan, China). The expression of BRD4, Ki‐67, and caspase‐3 were detected by immunohistochemistry in the specimens which were fxed in 10% neutral‐buffered formalin and subsequently embedded in paraffn. After a brief proteolytic digestion and peroxidase blocking of the tissue slides which were cut to a thickness of 4 µm previously, the slides were incubated overnight at 4°C with the anti‐BRD4 (1:1000, Abcam, USA), anti‐ki67, and anti‐caspase‐3 (All 1:100, Boster, China). In situ hybridization and IHC were scored in a semiquantitative manner. The scoring system was conducted as follows: 0 (<5%), 1 (5%‐30%), 2 (31%‐70%), and 3 (≥71%). Additionally, the staining intensity was stratified as follows: 0 (negative), 1 (weak), 2 (moderate), and 3 (strong). Based on the intensity score, the percentage of positive cells were multiplied to obtain the final immunoreactivity scores (IRS) for Ki‐67. Apoptosis ability in the tumor xenografts was also determined from the number of Caspase‐3‐positive cells.

### 2.15 Statistical analysis

MiR‐200a expression with clinical patient data was downloaded from the MSKCC (GSE21032) database (http://www.mskcc.org). Statistical analyses were performed with the Statistical Package of the Social Sciences software version 22.0 (SPSS, Inc, Armonk, NY, USA). Student's *t* test and one‐way ANOVA analysis were used to compare the significance of two groups. The Kaplan‐Meier method was performed to generate survival curves and Cox regression analysis was used for univariate and multivariate analyses. All experiments above were repeated three times and differences among groups in in vitro or in vivo studies were utilized as two‐tailed Student's *t* test. Data are presented as means and standard deviation (SD). A *P*‐value of <0.05 was considered statistically signifcant.

## 3 RESULTS

### 3.1 miR‐200a is specifically downregulated in CRPC and inversely associated with progression and poor prognosis of CRPC

**Figure 1:**
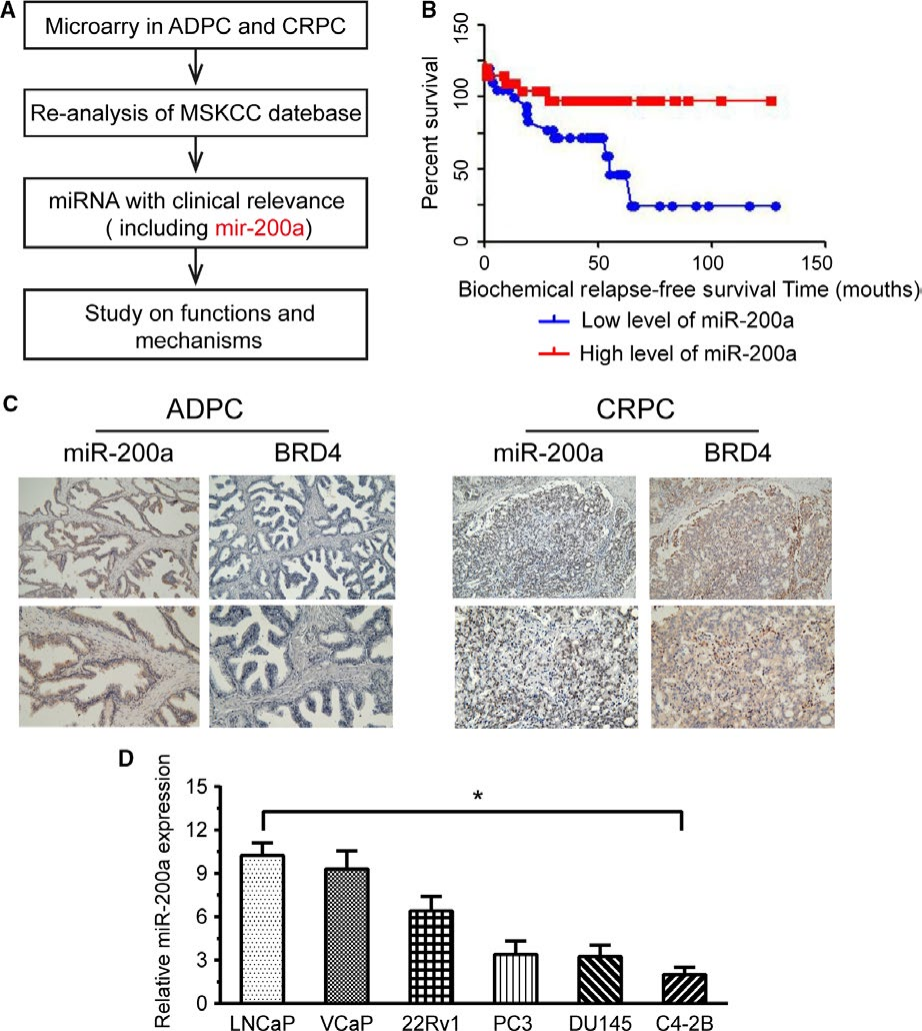
miR‐200a is specifically downregulated in castration‐resistant prostate cancer (CRPC). A, Experimental scheme. B, Kaplan‐Meier analysis of biochemical relapse‐free survival for 98 patients with prostate cancer. C, In situ hybridization and immunohistochemical staining on androgen‐dependent prostate cancer tissues and CRPC tissues. D, RT‐PCR was used to detect the expression level of miR‐200a in prostate cancer cell lines, including LNCaP, VCAP, 22Rv1, PC3, DU145, and C4‐2B. Results are representative of three independent experiments (*P* < 0.05)

In our previous microarray analysis, we totally detected 1646 miRNAs expressed in prostate cancer tissue.^19^ Among them, we found that 370 miRNAs were differentially expressed between ADPC and CRPC. From the microarray dataset, we discovered 167 miRNAs were downregulated in CRPC. As shown in Table 1, miRNAs including miR‐200a, miR‐205, miR‐1248, miR‐26a, and miR‐130a, were significantly decreased in CRPC in comparison to ADPC, among which miR‐200a was initially selected for further study (Figure 1A).

**Table 1:** Microarray analysis in ADPC and CRPC

High expressed miRNA	Normalized Intensity		CV		Log2(Ratio) ADPC/CRPC	*P*‐value ADPC/CRPC
	CRPC	ADPC	CRPC	ADPC		
MiR‐200a	101.407	1835.411	0.089	0.004	4.177	1.61E‐9
MiR‐205	128.736	4476.964	0.028	0.024	5.120	0
MiR‐1248	336.585	7428.018	0.114	0.010	4.463	1.44E‐8
MiR‐26a	467.479	9902.824	0.097	0.029	4.404	0.000001
MiR‐130a	92.776	1565.498	0.077	0.043	4.076	0.000003

ADPC, androgen‐dependent prostate cancer; CRPC, castration‐resistant prostate cancer; CV, coefficient of variation.

To validate whether the above conclusion is applicable to large number of clinical PCa samples, we conducted the reanalysis of the RNA sequencing data acquired from MSKCC PCa database (GSE21032). It is well‐known that biochemical recurrence of PCa refers to two consecutive serum PSA levels >0.2 μg/L after radical surgery or radiation treatment of prostate cancer. Therefore, the prostate cancer patient dataset used in this study was divided into miR‐200a‐high and miR‐200a‐low using the median expression level of miR‐200a as the cut‐off point. The Kaplan‐Meier method was used to analyze the relationship between miR‐200a expression and the time for biochemical recurrence after radical surgery of prostate cancer. The results showed that the biochemical relapse‐free survival (RFS) of patients in the miR‐200a‐low group was significantly shorter than that of the miR‐200a‐high group, *P* < 0.0001(Figure 1B). In order to clarify whether miR‐200a expression was associated with the outcome of PCa patients, we performed Cox regression analysis to identify variables of potential prognostic significance. The results suggested that miR‐200a expression (*P* = 0.032), Gleason score (GS) (*P* = 0.001), prostate‐specific antigen levels (PSA) (*P* = 0.002), and lymph node invasion (LNI) (*P* = 0.044) were independent prognostic factors for biochemical relapse‐free survival in patients with PCa. However, other factors such as seminal vesicle invasion (SVI), surgical margins (SMS), extracapsular extension (ECE), and pathological stage (pStage) were not predictive of prognosis (Table 2).

**Table 2:** Cox regression analysis of prognostic factors in PCa patients

Name	Hazard Ratio (HR)	95% CI for HR		*P* value
		Lower	Upper	
MiR‐200a	1.536	1.038	2.273	0.032
GS	8.896	2.452	32.268	0.001
PSA	1.057	1.021	1.094	0.002
LNI	0.044	0.006	0.333	0.044
Age	0.920	0.843	1.005	0.065
SMS	0.695	0.152	3.179	0.639
ECE	1.359	0.295	7.142	0.717
SVI	8.540	0.793	91.964	0.077
pStage	1.001	0.160	6.243	0.999

CI, confdence interval; ECE, extracapsular extension; GS, Gleason score; LNI, lymph node invasion; PSA, prostate‐specifc antigen; pStage, pathological.; SMS, surgical margins; SVI, seminal vesicle invasion.

All results from MSKCC database implied that miR‐200a‐low represents a poor prognostic factor of CRPC patient. Since miR‐200a has been reported to play the critical roles in multiple other malignancies, there is no report of miR‐200a involved in human CRPC. To verify the microarray results, we performed ISH staining on 10 ADPC tissues and 10 CRPC tissues with miR‐200a probe and we observed that miR‐200a expression was lower expression in CRPC compared with that in ADPC (Figure 1C). These data showed that miR‐200a may function as a tumor suppressor during PCa progression, and its expression level is associated with the transformation of ADPC to CRPC.

### 3.2 miR‐200a inhibits PCa cell proliferation, invasion, and facilitates apoptosis in vitro

**Figure 2:**
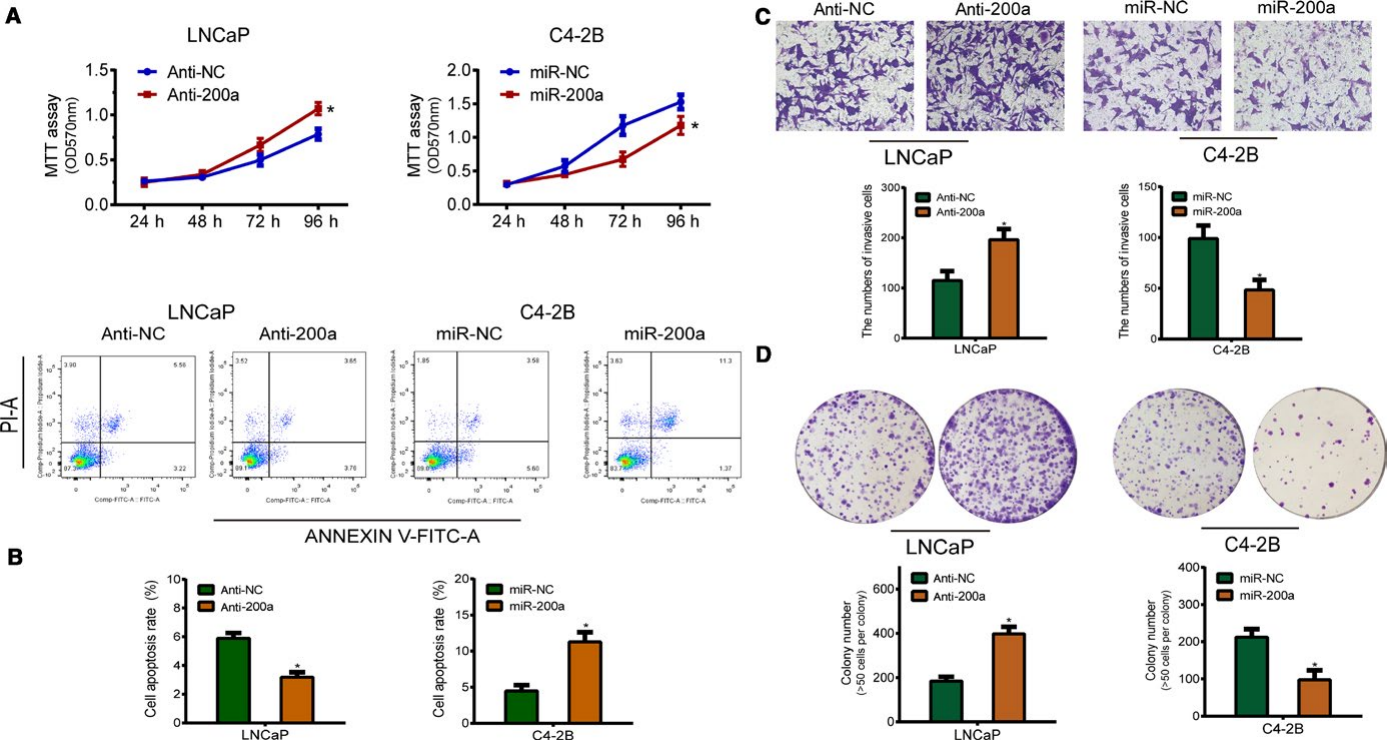
miR‐200a inhibits proliferation and invasion of prostate cancer cells, and facilitates apoptosis in vitro*. *A, Cell apoptosis assay analysis of the effect of miR‐200a expression alteration on cell apoptosis. B, MTT assay was performed to determine cell viability in LNCaP and C4‐2B cells (C) and (D) The prohibitive effect of miR‐200a on cell invasion and colony formation was assessed by Transwell and colony formation assays. Each bar represents the mean ± SD of three independent experiments. *P < 0.05

Quantitative real‐time PCR results showed that miR‐200a expression levels were significantly downregulated in C4‐2B cell lines in comparison to LNCaP cells (Figure 1D). Given the above results , C4‐2B and LNCaP cells were transfected with miR‐200a mimics or treated with a miR‐200a inhibitor. Cell proliferation was analyzed by MTT, transwell, and colony formation assays, and cell apoptosis was assayed by Annexin V‐FITC, and PI staining. qRT‐PCR demonstrated that transduction of miR‐200a mimics significantly upregulated the expression of miR‐200a in C4‐2B cells. In contrast, transfecting an anti‐miR‐200a into the miR‐200a high‐expressing LNCaP cells significantly downregulated miR‐200a expression in comparison to transfection of an anti‐miR‐NC. The upregulation of miR‐200a in C4‐2B cells showed a higher rate of apoptosis than the control group, whereas the downregulation of miR‐200a in LNCaP cells exhibited an opposite tendency in comparison to the controls (Figure 2A). Furthermore, the potential impact of miR‐200a on the cell proliferation, colony formation, and invasion of PCa was evaluated by performing MTT, colony formation, and transwell assays. As expected, overexpression of miR‐200a inhibited cell proliferation, colony formation, and invasion of C4‐2B cells in comparison to miR‐NC. Additionally, anti‐miR‐200a promoted cell proliferation, invasion, and colony formation of LNCaP cells, in comparison to the control anti‐miR‐NC (Figure 2B,C,D). In summary, these results indicate that, at the cellular level, miR‐200a suppresses PCa cell growth through inhibiting cell proliferation and reducing apoptosis.

### 3.3 Upregulation of miR‐200a suppresses the formation of prostate cancer xenograft tumors in vivo

**Figure 3:**
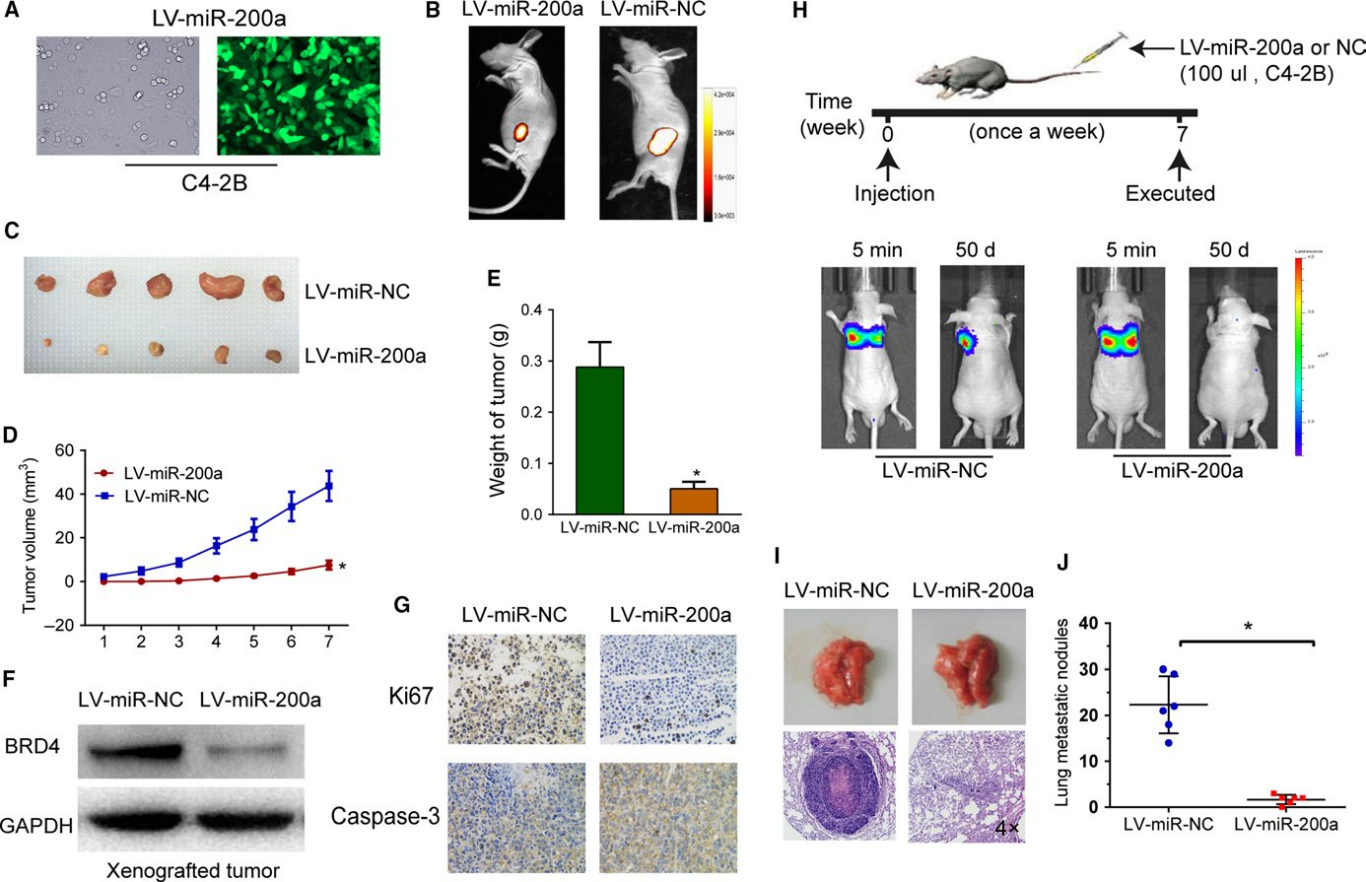
Upregulation of miR‐200a suppresses the formation of prostate cancer xenograft tumors in vivo*. *A, Fluorescence microscopy was used to assess the transfection efficiency of LV‐miR‐200a mimics. B and C, Representative images of tumors and subcutaneous tumors formed in nude mice (n = 5/group). D, Time‐dependent tumor formation growth curves. E, Histograms were used to describe the mean tumor weights of each group upon completion of the experiment. F, Immuohistochemical staining of Ki67 and activated caspase‐3 in the tumor xenografts. G, The endogenous bromodomain containing four (BRD4) expression levels in xenografts were detected by western blot analysis. GAPDH was used as an internal control. H, Representative bioluminescence imaging after tail vein injection of C4‐2B cells transfected with miR‐200a mimics or miR‐NC. I, Representative anatomical photos of lungs and representative lung tissue sections from nude mice (hematoxylin and eosin stain) were shown. J, Quantification of metastasis nodules of lungs after injecting C4‐2B cells transfected with miR‐200a mimics or miR‐NC (n = 5/group)

After subcutaneous injection of C4‐2B cells that transfected with preconstructed lentiviral expression vector (LV‐miR‐200a) and LV‐NC (control) into nude mice (Figure 3A), we further determined the tumor‐suppressive effects of miR‐200a. As shown in Figure 3B,C,D,E, upregulation of miR‐200a significantly suppressed tumor growth as verified by comparatively smaller tumor sizes and weights. We also showed by western blot analysis that miR‐200a‐overexpressing C4‐2B xenografts expressed higher levels of BRD4 protein in comparison to control xenografts (Figure 3F). Proliferation and apoptosis of the tumor xenografts were subsequently analyzed by immunohistochemical staining of the proliferation marker Ki67 and the apoptosis marker activated caspase‐3. The results revealed a reduced number of Ki67‐positive cells, and a significantly increased number of caspase‐3‐positive cells in miR‐200a‐overexpressing C4‐2B tumor xenografts (Figure 3G)We then measured the effect of miR‐200a on tumor metastasis in vivo, by injecting C4‐2B cells transfected with miR‐200a or NC into the tail veins of nude mice. Bioluminescence imaging demonstrated that miR‐200a suppressed metastasis of C4‐2B cells to the lungs (Figure 3H). After a 7‐week monitoring period, gross anatomical observation of the lungs revealed that the number of metastatic nodes was markedly decreased in the nude mice injected with C4‐2B cells transfected with miR‐200a mimics (Figure 3I,J). These results highlight the tumor‐suppressive effects of miR‐200a in prostate cancer. The aforementioned results illustrate that miR‐200a inhibits tumor formation in vivo.

### 3.4 BRD4 is a direct target of miR‐200a and the biological effect of miR‐200a is mediated by AR signaling in PCa

**Figure 4:**
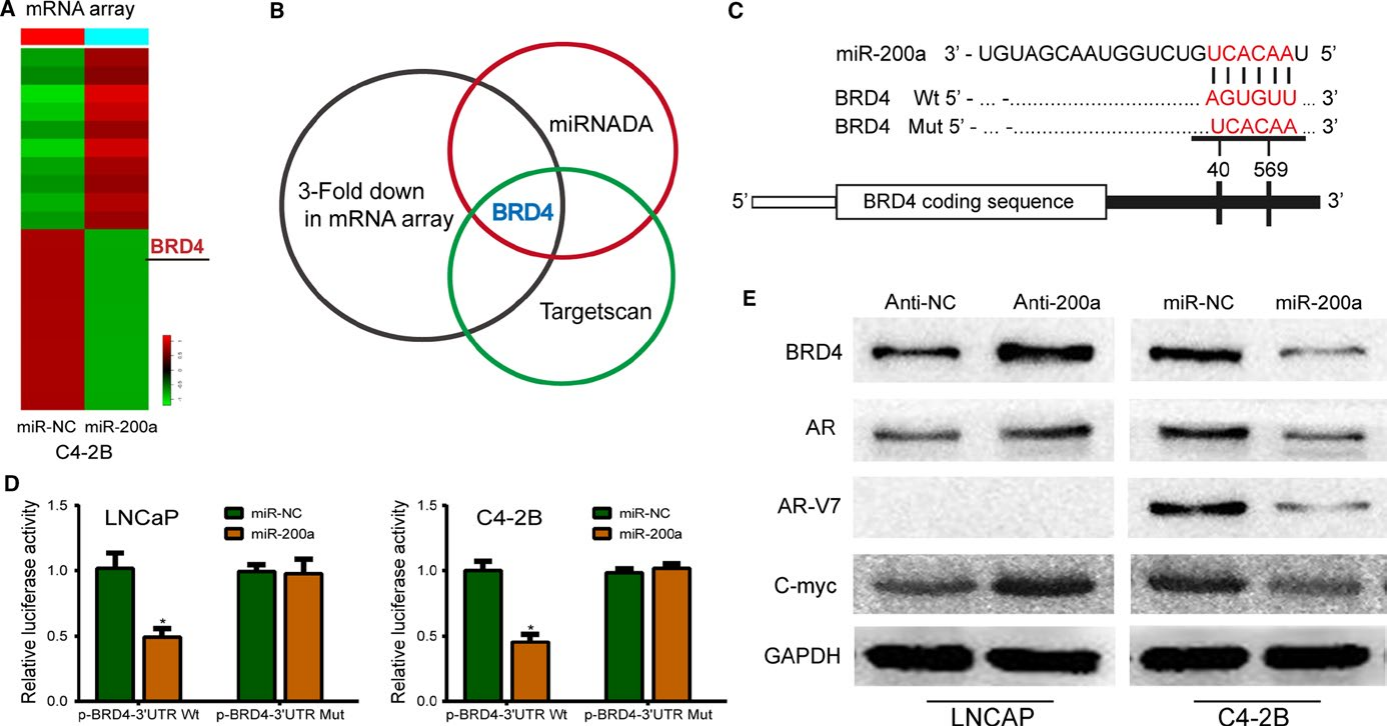
Bromodomain containing protein 4 (BRD4 is a direct target of miR‐200a and the biological function of miR‐200a is mediated by AR signaling in prostate cancer. A, Analysis of the RNA‐based microarray following miR‐200a overexpression. B, Overlap of miRNA target bioinformatic prediction methods, upregulation of miR‐200a induced knockdown mRNAs in conjunction with miRanda analysis. BRDR4 was found to be the intersection point of these datasets. C, The alignment of the corresponding mutated sequences within the BRD4 3′‐UTR for miR‐200a. D, Luciferase report assay was carried out by cotransfecting miR‐200a mimics or miR‐NC and psi‐CHECK‐BRD4‐WT, or psi‐CHECK‐BRD4‐MUT into LNCaP and C4‐2B cells. The relative firefly luciferase activity was normalized and measured 48 h after transfection. Each bar represents the mean ± SD of three independent experiments. *P < 0.05. E, Western blot assay showed the comparison of relevant protein expression levels. GAPDH was used as an internal control

After performing affymetrix human gene expression array analysis, Target prediction program (TargetScan and miRanda) software, *BRD4* was identified as a top candidate target gene of miR‐200a (Figure 4A,B). To further investigate the relationship between BRD4 and miR‐200a in PCa, we performed ISH and IHC analysis of 10 ADPC tissues and 10 CRPC tissues, using a miR‐200a probe and an anti‐BRD4 antibody. We observed that BRD4 expression was inversely correlated with miR‐200a level (Figure 1C). Similarly, western blotting further indicated that BRD4 expression in C4‐2B miR‐200a‐overexpressing xenografted tumors was higher in comparison to controls. BRD4 has been demonstrated to be a key component of the AR signaling pathway. Therefore, we hypothesized that AR signaling may be a major mediator of the biological function of miR‐200a in PCa. To verify whether *BRD4* is a functional target of miR‐200a, a luciferase reporter assay was carried out by cotransfecting miR‐200a mimics and miR‐NC with psi‐CHECK‐BRD4‐WT (harbors the wild‐type miR‐200a binding site in the BRD4 3′‐UTR downstream of the firefly luciferase gene), or psi‐CHECK‐BRD4‐MUT (contains a mutated miR‐200a binding site in the BRD4 3′‐UTR) into LNCaP and C4‐2B cells. In this assay, relative luciferase activity was markedly reduced in both LNCaP and C4‐2B cells cotransfected with psi‐CHECK‐NKD1‐MUT luciferase reporter and miR‐200a mimics in comparison to NC control cells. In contrast, the expression of the luciferase reporter containing a mutated sequence of the BRD4 binding site (psi‐CHECK‐BRD4‐MUT) was not affected by cotransfection with miR‐200a mimics (Figure 4C,D), which further demonstrates that *BRD4 *is a direct target of miR‐200a. Moreover, western blot analysis revealed that silencing of miR‐200a not only enhanced enrichment of BRD4, but increased expression of other positive regulators of AR signaling including AR and C‐myc as well. Notably, LNCaP cells have been demonstrated to not express AR‐V7 protein, which accounts for the blank protein band in the location of AR‐V7. Correspondingly, aforementioned regulatory molecules as well as AR‐V7 concerning AR signaling were reduced after overexpressing miR‐200a in C4‐2B cells (Figure 4E).

### 3.5 BRD4 can reverse miR‐200a‐mediated biological effects in PCa cells

**Figure 5:**
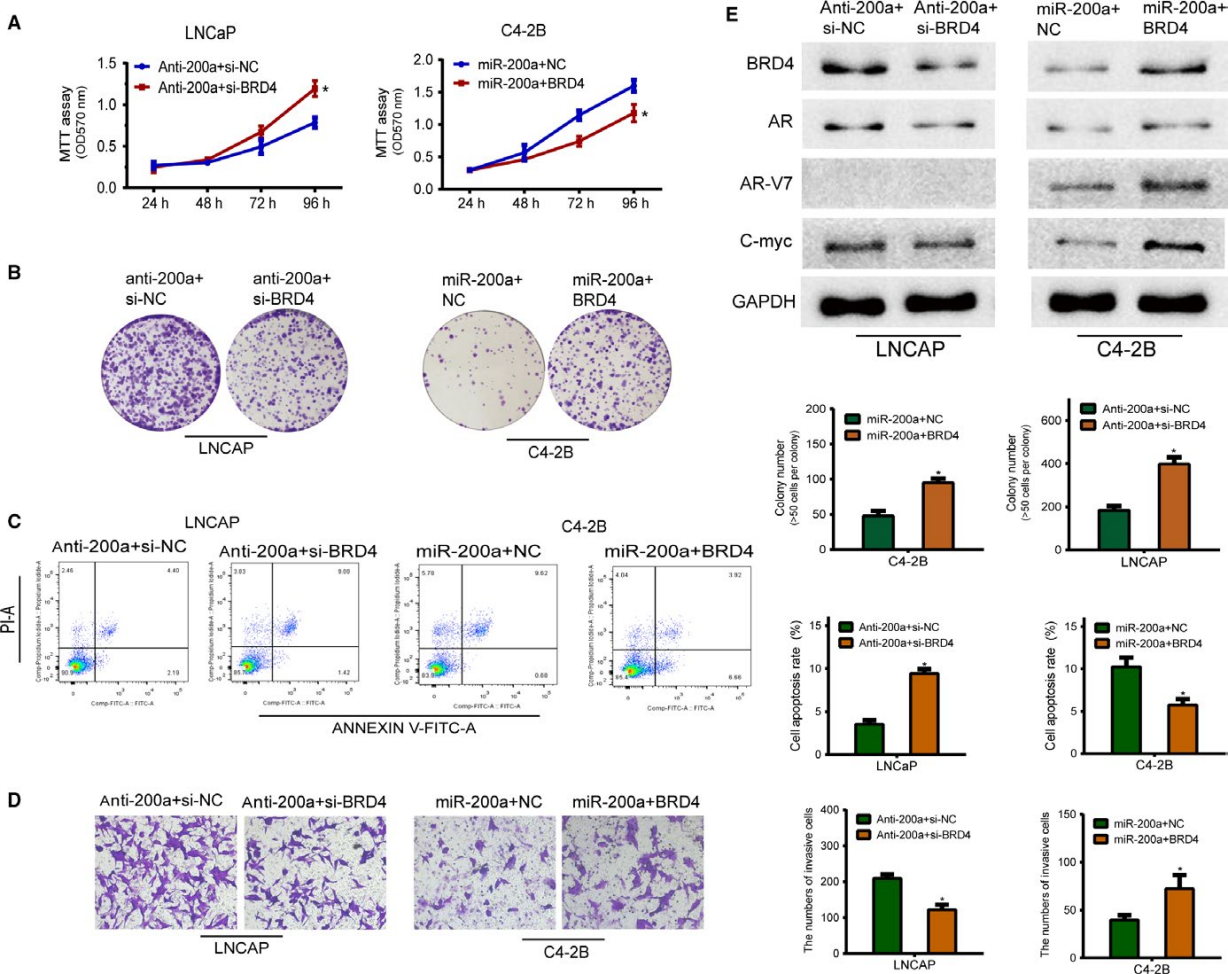
Bromodomain containing protein 4 (BRD4) is a crucial mediator reversing miR‐200a‐mediated biological effects in prostate cancer cells. A, B and D, The negative or positive effect of perturbing the expression of miR‐200a on cell proliferation, colony formation, and invasion was reversed by the high expression of BRD4 or by BRD4 inhibitor treatment. C, miR‐200a overexpression or si‐BRD40‐induced apoptosis. Each bar represents the mean ± SD of three independent experiments. *P < 0.05. E, The expression levels of BRD4 protein and AR signaling pathway‐related proteins were analyzed by western blotting. GAPDH was used as an internal control

We investigated whether BRD4 may functionally reverse the miR‐200a‐mediated biological effects in PCa cells. LNCaP and C4‐2B cells with anti‐miR‐200a and miR‐200a mimics, were further cotransfected with siBRD4 or a BRD4‐overexpressing vector (BRD4‐pc). Furthermore, double‐infected LNCaP and C4‐2B cells were then evaluated by MTT, cell apoptosis, transwell, and colony formation assays. These assays demonstrated that, in comparison to NC controls, knockdown of BRD4 by transfecting siBRD4 moderately attenuated the promotion of cell proliferation, colony formation, and invasion of LNCaP cells induced by downregulation of miR‐200a levels. Additionally, overexpression of BRD4 reversed the inhibitory effects on cell proliferation, colony formation, and invasion of C4‐2B cells induced by upregulation of miR‐200a (Figure 5A,B,D).

Also, knockdown of BRD4 resulted in a higher level of apoptosis in LNCaP cells transfected with anti‐miR‐200a, in comparison to LNCaP cells cotransfected with anti‐miR‐200a and NC. On the contrary, C4‐2B cells transfected with miR‐200a mimics and BRD4 showed a reduction in apoptosis in comparison to cells cotransfected with miR‐200a mimics and NC (Figure 5C). Western blot assay also demonstrated that knockdown of BRD4 significantly antagonized the enrichment of BRD4, AR, and C‐myc induced by anti‐miR‐200a in LNCaP cells, and enhanced the expression level of BRD4, AR, AR‐V7, and C‐myc inhibited by the overexpression of miR‐200a in C4‐2B cells (Figure 5E). Therefore, our data strongly suggest that BRD4 is functionally involved in miR‐200a‐mediated suppression in PCa cells.

## 4 DISCUSSION

miRNAs have been extensively studied over the past two decades, and their involvement in virtually all cellular processes by regulating gene expression has been well demonstrated. Therefore, the aberrant expression of these short nucleotide sequences can serve tumor‐suppressive or protumorigenic roles in many malignant tumors, including prostate cancer.^20^ In this study, we investigated the molecular function of miR200a and identified novel gene targets of miR‐200a in PCa. We found that upregulation of miR‐200a significantly inhibited cell proliferation and migration, and promoted apoptosis of PCa cell lines. Moreover, *BRD4 *was identified as a novel gene target of miR‐200a, and a mediator of miR‐200a oncogenic activity through AR signaling.

The first report concerning miR‐200a showed its high expression in olfactory tissues.^21^ Subsequently, its involvement in human disease was demonstrated with downregulated miR‐200a involvement in cellular biological processes, including cell proliferation, and apoptosis. Moreover, it has been reported that miR‐200a is related to the occurrence and development of endometrial cancer, breast cancer, and esophageal cancer by targeting genes, such as *PTEN, EPHA2,* and *CRMP‐1*.^15^, ^22^, ^23^ In prostate cancer, it has been reported that miR‐200b/a inhibits PCa cell growth and invasion by downregulating ERG expression.^24^ Bian et al demonstrated that miR‐200a is involved in the oncogenesis and migration of CRPC.^25^ Another study showed that miR‐200a is overexpressed in prostate cancer and may serve as a predictive biomarker of RFS.^26^ miRNAs, such as miR‐125b, miR‐146a, miR‐744, and miR‐34a, have also been shown to function either as an oncogene or as a tumor suppressor in PCa.^12^, ^27^, ^28^, ^29^ However, the underlying molecular mechanisms of the biological functions of miR‐200a remain unknown.

In our current study, we discovered that miR‐200a levels are lower in human CRPC specimens than in human ADPC samples. We also demonstrated that miR‐200a can suppress cell proliferation, colony formation, and invasion of CRPC in vitro*,* as well as enhance xenograft tumor growth in vivo. Therefore, we surmised that miR‐200a behaves as an anti‐oncogenic factor in the progression of ADPC to CRPC.

The differential target genes regulated by miR‐200a are possibly responsible for the protumorigenic effects of miR‐200a. We used a luciferase reporter assay to demonstrate that *BRD4* is a target gene of miR‐200a. BRD4, a member of the BET (Bromodomain and extraterminal domain) family, is a transcriptional regulator in mitotic cells and plays a crucial role in cancers. BET proteins bind to the chromosome and regulated gene expression by recognizing the acetyl‐lysine residues of histones or by interacting with other transcription factors, such as members of the transcription elongation complex.^30^ Aside from its essential role in normal cell cycle, differentiation, and development, BRD4 has also been demonstrated to participate in various biological processes in tumor cells, including cell invasion, migration, proliferation, and EMT, by acting as an oncogene.^31^ A growing body of evidence has documented that BRD4 can serve as a prognostic factor of bladder urinary epithelial carcinoma, serves as a treatment target for acute myelogenous leukemia, and predicts the survival of breast cancer patients.^32^, ^33^, ^34^ However, the molecular role and clinical relevance of BRD4 in PCa remains unclear. Here, we detected that BRD4 was upregulated in PCa tissues. In vitro assays indicated that *BRD4* downregulation inhibited the proliferation of PCa tumor cells. Additionally, xenograft tumor models further showed that the knockdown of *BRD4* significantly suppressed tumor growth in vivo. These findings demonstrated that BRD4 functions as a protumorigenic factor in PCa progression.

**Figure 6:**
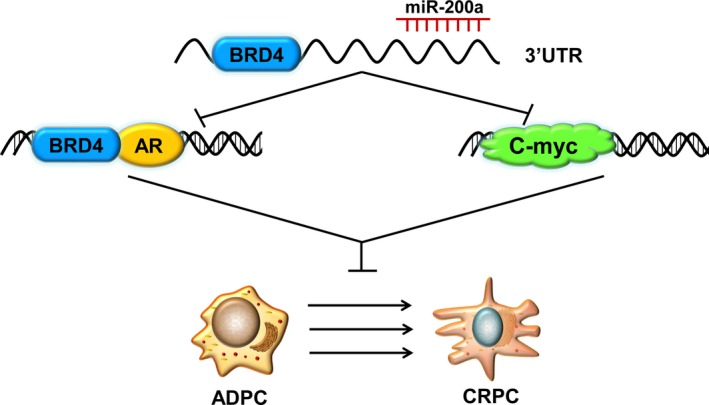
Schematic diagram of miR‐200a/BRD4/AR signaling pathway

Previous studies have demonstrated that BRD4 can promote the transcriptional activities of oncogenic factors in prostate cancer by physically interacting with the N‐terminal domain of androgen receptor (AR), which is a crucial element of the AR signaling pathway. The progression of ADPC to CRPC is characterized by the abnormal activation of AR signaling and overexpression of AR target genes, such as PSA, the expression of which is tightly association with PCa progression. To further verify the relationship between miR‐200a and AR signaling, we found that upregulation of miR‐200a in LNCaP cell lines transfected with miR‐200a mimics showed a significant downregulation of BRD4, AR, and c‐myc based on western blotting. With respect to BRD4, we found that after transfecting these LNCaP cells with a BRD4‐overexpressing vector, BRD4 expression was significantly upregulated in miR‐200a‐upregulated LNCaP cells by qRT‐PCR and western blotting in comparison to NC control cells. Therefore, our data suggest that BRD4 is functionally involved in miR‐200a‐mediated suppression in PCa cells. Additionally, miR‐200a acts as a negative regulator of BRD4/AR signaling (Figure 6).

Studies have demonstrated that BET inhibitors (BETi) such as JQ1 can competitively bind to the amino terminal bromodomains of BRD4, leading to the replacement of BRD4 from active chromatin. As a result, RNA polymerase II is removed from active chromatin, ultimately altering the expression of target genes, such as *FOS, Jun, *and* c‐myc,* which are significantly involved in AR signaling in CRPC.^35^, ^36^ Additionally, it has been reported that BETi can directly modulate *c‐myc* gene expression and subsequently abrogate AR signaling and the progression of CRPC.^36^, ^37^ Therefore, inhibiting the positive modulator of BRD4 represents a novel strategy to control the aberrant activation of AR signaling. Until now, a number of bromodomain inhibitors, including JQ1 and I‐BET, have been developed and have shown promising outcomes in preclinical and early clinical trials.^36^, ^38^ Our study has uncovered a novel anti‐tumorigenic role for miR‐200a in the progression of CRPC, which is mediated by disrupting BRD4/AR signaling.

In summary, this study characterized the role of miR‐200a and its anti‐tumorigenic effect of attenuating the progression of ADPC to CRPC. Most importantly, we identified the functional regulation of BRD4‐AR signaling by miR‐200a, which associates with patient outcomes in aggressive prostate cancer. Therefore, the downstream gene regulation by miR‐200a/BRD4 should be investigated further to foster the development of novel anticancer therapies for patients with CRPC.

## CONFLICT OF INTEREST

The authors have declared that there are no conflicts of interest.
